# Integrated Resistance Analysis of CERTAIN-1 and CERTAIN-2 Studies in Hepatitis C Virus-Infected Patients Receiving Glecaprevir and Pibrentasvir in Japan

**DOI:** 10.1128/AAC.02217-17

**Published:** 2018-01-25

**Authors:** Preethi Krishnan, Gretja Schnell, Rakesh Tripathi, Jill Beyer, Thomas Reisch, Tatyana Dekhtyar, Michelle Irvin, Wangang Xie, Bo Fu, Margaret Burroughs, Rebecca Redman, Hiromitsu Kumada, Kazuaki Chayama, Christine Collins, Tami Pilot-Matias

**Affiliations:** a Research and Development, AbbVie Inc., North Chicago, Illinois, USA; b Department of Hepatology, Toranomon Hospital, Tokyo, Japan; c Department of Gastroenterology and Metabolism, Hiroshima University, Hiroshima, Japan

**Keywords:** HCV, NS5A inhibitor, glecaprevir, pibrentasvir, protease inhibitors

## Abstract

Glecaprevir and pibrentasvir are hepatitis C virus (HCV) pangenotypic inhibitors targeting NS3/4A protease and NS5A, respectively. This once-daily, fixed-dose combination regimen demonstrated high sustained virologic response 12 weeks postdosing (SVR_12_) rates in CERTAIN-1 and CERTAIN-2 studies in Japanese HCV-infected patients, with a low virologic failure rate (1.2%). There were no virologic failures among direct-acting antiviral (DAA)-treatment-naive genotype 1a (GT1a) (*n* = 4)-, GT1b (*n* = 128)-, and GT2 (*n* = 97)-infected noncirrhotic patients treated for 8 weeks or among GT1b (*n* = 38)- or GT2 (*n* = 20)-infected patients with compensated cirrhosis treated for 12 weeks. Two of 33 DAA-experienced and 2 of 12 GT3-infected patients treated for 12 weeks experienced virologic failure. Pooled resistance analysis, grouped by HCV subtype, treatment duration, prior treatment experience, and cirrhosis status, was conducted. Among DAA-naive GT1b-infected patients, the baseline prevalence of NS3-D168E was 1.2%, that of NS5A-L31M was 3.6%, and that of NS5A-Y93H was 17.6%. Baseline polymorphisms in NS3 or NS5A were less prevalent in GT2, with the exception of the common L/M31 polymorphism in NS5A. Among DAA-experienced GT1b-infected patients (30/32 daclatasvir plus asunaprevir-experienced patients), the baseline prevalence of NS3-D168E/T/V was 48.4%, that of NS5A-L31F/I/M/V was 81.3%, that of the NS5A P32deletion was 6.3%, and that of NS5A-Y93H was 59.4%. Common baseline polymorphisms in NS3 and/or NS5A had no impact on treatment outcomes in GT1- and GT2-infected patients; the impact on GT3-infected patients could not be assessed due to the enrollment of patients infected with diverse subtypes and the limited number of patients. The glecaprevir-pibrentasvir combination regimen allows a simplified treatment option without the need for HCV subtyping or baseline resistance testing for DAA-naive GT1- or GT2-infected patients. (The CERTAIN-1 and CERTAIN-2 studies have been registered at ClinicalTrials.gov under identifiers NCT02707952 and NCT02723084, respectively.)

## INTRODUCTION

Hepatitis C virus (HCV) is an enveloped, single-stranded, positive-sense RNA virus in the Flaviviridae family that infects approximately 71 million people worldwide (1). It is estimated that 1.5 million people in Japan are infected with HCV (2). Globally, 6 distinct HCV genotypes (GTs) and 67 subtypes have been characterized (3). In Japan, approximately 70% of HCV infections are GT1b, 25 to 30% are GT2, and 2% are other GTs (GT3, -4, -5, or -6) (4–7). In contrast to the United States and many parts of Europe, very few HCV-infected patients (<1% of GT1-infected patients) are infected with GT1a in Japan (8), and the subtype diversity in GT2 is mostly limited to GT2a and GT2b (2).

Therapy for HCV was improved considerably with the availability of several interferon (IFN)-free direct-acting antiviral (DAA) regimens. In Japan, IFN-free DAA regimens, including daclatasvir plus asunaprevir with or without beclabuvir, ledipasvir-sofosbuvir, ombitasvir-paritaprevir-ritonavir, and elbasvir plus grazoprevir with or without ribavirin (RBV), are available for HCV GT1 treatment (9–11). Sofosbuvir plus RBV and ombitasvir-paritaprevir-ritonavir plus RBV were the IFN-free regimens available for the treatment of patients infected with HCV GT2, while sofosbuvir plus RBV was recommended for the treatment of HCV GT3 to GT6 in Japan according to Japan Society of Hepatology (JSH) 2016 guidelines for the management of hepatitis C virus infection (10, 12, 13). The approved and recommended regimens were not equally effective across all HCV genotypes and subpopulations. Additional limitations of several of the above-listed approved regimens included the requirement for the inclusion of RBV in certain populations, significant drug-drug interactions, limited options for patients with renal insufficiency, reduced efficacy in patients with baseline amino acid polymorphisms associated with reduced susceptibility to HCV nonstructural viral protein 3/4A (NS3/4A) protease inhibitors (PIs) or NS5A inhibitors, and limited options for patients who had failed DAA-containing treatment regimens (10).

Glecaprevir (formerly ABT-493, identified by AbbVie and Enanta), an NS3/4A PI, and pibrentasvir (formerly ABT-530), an NS5A inhibitor, are next-generation HCV inhibitors. Both drugs have potent *in vitro* antiviral activities against GT1 to GT6, with little or no loss of potency against common single resistance-associated amino acid substitutions (14, 15). Additive or synergistic *in vitro* anti-HCV activity has been demonstrated with the combination of glecaprevir and pibrentasvir (14). Glecaprevir and pibrentasvir, coformulated into a fixed-dose combination tablet, were evaluated as a pangenotypic regimen in 8 phase 2 and 3 clinical studies across North America, Europe, and the rest of the world (ROW) (Australia, Chile, Israel, South Korea, New Zealand, South Africa, and Taiwan) (16). Among 2,256 HCV GT1- to GT6-infected patients without cirrhosis or with compensated cirrhosis who were treatment naive or treatment experienced (to pegylated IFN [pegIFN], RBV, and/or sofosbuvir) and treated with glecaprevir-pibrentasvir for 8, 12, or 16 weeks, fewer than 1% (22/2,256) of patients experienced virologic failure (16). The regimen has recently received marketing approvals in Europe, the United States, and Canada for treatment durations of 8 weeks in noncirrhotic treatment-naive GT1- to GT6-infected patients; 8 weeks in noncirrhotic treatment-experienced GT1-, GT2-, GT4-, GT5-, or GT6-infected patients; 12 weeks in treatment-naive or treatment-experienced GT1-, GT2-, GT4-, GT5-, or GT6-infected patients with compensated cirrhosis; 12 weeks in treatment-naive GT3-infected patients with compensated cirrhosis; and 16 weeks in treatment-experienced GT3-infected patients irrespective of cirrhosis status (17, 18).

The glecaprevir-pibrentasvir regimen is approved in the United States and Canada for the treatment of PI-experienced patients without prior experience with an NS5A inhibitor (12-week treatment duration) or NS5A inhibitor-experienced patients without prior experience with a PI (16-week treatment duration). The SVR_12_ rates among GT1-infected patients treated with the glecaprevir-pibrentasvir regimen who were PI experienced/NS5A inhibitor naive or NS5A inhibitor experienced/PI naive were 92% (23/25) and 94% (16/17), respectively (17).

The safety and efficacy of glecaprevir-pibrentasvir in HCV-infected patients in Japan was evaluated in two phase 3 clinical studies, CERTAIN-1 and CERTAIN-2 (19–21). Treatment-naive or (peg)IFN with or without RBV-experienced (but DAA treatment-naive) GT1- and GT2-infected noncirrhotic patients received glecaprevir-pibrentasvir for 8 weeks, and patients with compensated cirrhosis received 12 weeks of treatment. GT3- to GT6-infected patients and DAA treatment-experienced GT1- or GT2-infected patients without cirrhosis or with compensated cirrhosis received 12 weeks of treatment. High SVR_12_ rates were observed for all patient populations and genotypes (Tables 1 and 2). The glecaprevir-pibrentasvir regimen recently received marketing approval in Japan for a treatment duration of 8 weeks in DAA treatment-naive GT1- and GT2-infected patients without cirrhosis, including those with chronic kidney disease, and for a treatment duration of 12 weeks for patients infected with GT3 to GT6, patients with compensated cirrhosis, and those not cured with a previous DAA treatment.

**TABLE 1:** SVR_12_ rates by HCV subtype, treatment duration, prior treatment experience, and cirrhosis status in DAA-naive patients^*f*^

HCV subtype^*a*^	SVR_12_ (%) (no. of patients with response/total no. of patients enrolled)					
	Without cirrhosis				With compensated cirrhosis, 12 wk	
	8 wk		12 wk		TN	IFN-TE
	TN^*b*^	IFN-TE^*c*^	TN	IFN-TE		
1a	100 (3/3)	100 (1/1)	X	X		
1b	100 (93/93)	97.1 (34/35)^*d*^	X	X	100 (26/26)	100 (12/12)
2a	96.5 (55/57)^*d*^	100 (11/11)	X	X	100 (4/4)	100 (6/6)
2b	100 (21/21)	100 (6/6)	X	X	100 (9/9)^*e*^	100 (1/1)
2*	100 (2/2)		X	X		
3a	X	X	100 (3/3)	100 (3/3)	100 (1/1)	
3b	X	X	100 (1/1)	50.0 (1/2)	100 (1/1)	
3k	X	X	0 (0/1)			

a HCV subtype was determined by phylogenetic analysis of NS3/4A and/or NS5A sequences. In the absence of phylogenetic data (4 treatment-naive GT1b- and 2 treatment-naive GT2*-infected patients), subtype designation was done by LiPA 2.0 assay.

b Includes 2 GT1b-infected, 1 GT2a-infected, 2 GT2b-infected, and 2 GT2 (with an undetermined subtype)-infected SVR_12_-achieving patients with severe renal impairment.

c Includes 1 GT1b- and 2 GT2a-infected SVR_12_-achieving patients with severe renal impairment.

d Patients not achieving SVR_12_ were lost to follow-up or prematurely discontinued treatment.

e Includes 2 GT2b-infected SVR_12_-achieving patients with severe renal impairment.

f X indicates that patients with these HCV genotypes were excluded per enrollment criteria. * indicates that the HCV subtype was unable to be determined. IFN-TE, treatment experienced with an interferon-containing regimen but DAA naive; TN, DAA treatment naive.

**TABLE 2:** SVR_12_ rates by HCV subtype, prior DAA treatment experience, and cirrhosis status in DAA-experienced patients treated for 12 weeks

HCV subtype^*a*^	SVR_12_ (%) (no. of patients with response/total no. of patients enrolled)			
	DAA experienced without cirrhosis			DAA experienced with compensated cirrhosis (PI experienced and NS5A inhibitor experienced)
	PI experienced and NS5A inhibitor naive	PI experienced and NS5A inhibitor experienced	Nucleotide NS5B polymerase inhibitor experienced	
1b	100 (2/2)	96.2 (25/26)		75.0 (3/4)
2a			100 (1/1)	

a HCV subtype was determined by phylogenetic analysis of NS3/4A and/or NS5A sequences.

Pooled data from efficacy and resistance analyses of the CERTAIN-1 and CERTAIN-2 studies is presented. Baseline sequencing of HCV NS3/4A and NS5A was conducted by next-generation sequencing (NGS) on all available baseline samples to assess the prevalence and impact of baseline polymorphisms on treatment outcomes. Analyses were integrated across the two studies by HCV subtype, prior DAA treatment experience, treatment duration, and cirrhosis status. Baseline polymorphisms and treatment-emergent substitutions in patients experiencing virologic failure were also evaluated. The prevalences of baseline polymorphisms were compared by geographic region, using sequence data from non-Japan (referred to as overseas regions) phase 2 and 3 studies with the glecaprevir-pibrentasvir regimen.

## RESULTS

### Efficacy of glecaprevir-pibrentasvir in analyses of pooled data from the CERTAIN-1 and CERTAIN-2 clinical studies.

The SVR_12_ rates by HCV subtype, prior treatment experience, treatment duration, and cirrhosis status are shown in Tables 1 and 2. Among DAA treatment-naive GT1- and GT2-infected patients without cirrhosis treated for 8 weeks, including 10 patients with severe renal impairment, there were no virologic failures, and the SVR_12_ rates were 99.2% (131/132; 1 patient was lost to follow-up) and 97.9% (95/97; 1 patient prematurely discontinued the study drug, and 1 patient was lost to follow-up) for GT1- and GT2-infected patients, respectively (Table 1). SVR_12_ rates of 100% were observed among GT1 (*n* = 38)- and GT2 (*n* = 20)-infected patients with compensated cirrhosis treated for 12 weeks, including 2 GT2b-infected patients with severe renal impairment. SVR_12_ was achieved in 83.3% (10/12; 2 patients experienced posttreatment relapse) of patients with GT3 HCV infection treated for 12 weeks. The CERTAIN-1 study enrolled 33 patients with HCV GT1 or GT2 infection who failed at least 1 previous DAA treatment, and SVR_12_ was achieved in 93.9% (31/33) of patients in this population (Table 2). None of the patients enrolled in the two CERTAIN studies were infected with HCV GT4, GT5, or GT6.

### Analysis of baseline polymorphisms in NS3 and NS5A in DAA-naive patients.

The prevalence of baseline polymorphisms relative to the subtype-specific reference sequence was evaluated for 4 GT1a-, 162 GT1b-, 78 GT2a-, 37 GT2b-, 7 GT3a-, 4 GT3b-, and 1 GT3k-infected DAA-naive patients grouped by HCV subtype, prior treatment experience, and cirrhosis status (Table 3). The prevalences of baseline polymorphisms were similar across treatment-naive and (peg)IFN (with or without RBV)-experienced patients without cirrhosis or with compensated cirrhosis for all genotypes. Among GT1b-infected patients with an available baseline sequence (*n* = 162), the prevalences of polymorphisms at amino acid position 56 or 80 (mostly Q80L) in NS3 were high, 38.9% and 18.5%, respectively, while polymorphisms at position 168 were rare. In NS5A, the prevalence of Y93H was 18.0%, and the prevalence of polymorphisms at position 24, 28, 30, 58, 92, or 93 ranged between 7.5% and 11.8%, while L31M was less frequent.

**TABLE 3:** Prevalence of baseline polymorphisms among DAA-naive patients

HCV subtype^*a*^	Target	Baseline polymorphism^*b*^	% of patients with baseline polymorphism (no. of patients with polymorphism/total no. of patients sequenced)				
			Total	Without cirrhosis^*c*^		With compensated cirrhosis^*c*^	
				TN^*d*^	IFN-TE^*d*^	TN^*d*^	IFN-TE^*d*^
1a	NS3	Q80K/L	50.0 (2/4)	66.7 (2/3)			
		Any	50.0 (2/4)	66.7 (2/3)	(0/1)		
	NS5A	M28V	25.0 (1/4)		100 (1/1)		
		Q30H	25.0 (1/4)	33.3 (1/3)			
		Y93F	25.0 (1/4)	33.3 (1/3)			
		Any	50.0 (2/4)	33.3 (1/3)	100 (1/1)		
1b	NS3	V36L	0.6 (1/162)			3.8 (1/26)	
		T54S	3.1 (5/162)	2.2 (2/89)	5.7 (2/35)	3.8 (1/26)	
		V55I	1.9 (3/162)	1.1 (1/89)		7.7 (2/26)	
		Y56F	38.9 (63/162)	43.8 (39/89)	31.4 (11/35)	30.8 (8/26)	41.7 (5/12)
		Q80K/L/R	18.5 (30/162)	15.7 (14/89)	25.7 (9/35)	23.1 (6/26)	8.3 (1/12)
		D168E	1.2 (2/162)	1.1 (1/89)		3.8 (1/26)	
		Any	50.6 (82/162)	49.4 (44/89)	51.4 (18/35)	53.8 (14/26)	50.0 (6/12)
	NS5A	Q24K/R	8.1 (13/161)	6.8 (6/88)	5.7 (2/35)	15.4 (4/26)	8.3 (1/12)
		L28M	8.1 (13/161)	6.8 (6/88)	5.7 (2/35)	15.4 (4/26)	8.3 (1/12)
		R30H/L/Q	11.8 (19/161)	13.6 (12/88)	5.7 (2/35)	19.2 (5/26)	
		L31M	3.7 (6/161)	3.4 (3/88)	2.9 (1/35)	7.7 (2/26)	
		P58Q/R/S/T	7.5 (12/161)	9.1 (8/88)	8.6 (3/35)	3.8 (1/26)	
		A92E/P/T/V	8.1 (13/161)	5.7 (5/88)	11.4 (4/35)	3.8 (1/26)	25.0 (3/12)
		Y93H	18.0 (29/161)	20.5 (18/88)	14.3 (5/35)	19.2 (5/26)	8.3 (1/12)
		Any	42.9 (69/161)	43.2 (38/88)	37.1 (13/35)	50.0 (13/26)	41.7 (5/12)
2a	NS3	L36I/M	4.0 (3/75)	5.3 (3/57)			
		Y56F	4.0 (3/75)	3.5 (2/57)			20.0 (1/5)
		D168E	1.3 (1/75)	1.8 (1/57)			
		Any	9.3 (7/75)	10.5 (6/57)	(0/11)	(0/2)	20.0 (1/5)
	NS5A	T24A/S	6.4 (5/78)	5.3 (3/57)		25.0 (1/4)	16.7 (1/6)
		F28C/L	2.6 (2/78)	3.5 (2/57)			
		L31M	92.3 (72/78)	93.0 (53/57)	100 (11/11)	75.0 (3/4)	83.3 (5/6)
		P58S	7.7 (6/78)	7.0 (4/57)		25.0 (1/4)	16.7 (1/6)
		C92N/S	3.8 (3/78)	5.3 (3/57)			
		Any	94.9 (74/78)	96.5 (55/57)	100 (11/11)	75.0 (3/4)	83.3 (5/6)
2b	NS3	Y56F	5.4 (2/37)		16.7 (1/6)	11.1 (1/9)	
		Any	5.4 (2/37)	(0/21)	16.7 (1/6)	11.1 (1/9)	(0/1)
	NS5A	L28F	16.2 (6/37)	14.3 (3/21)		22.2 (2/9)	100 (1/1)
		M31I/L/V	16.2 (6/37)	23.8 (5/21)	16.7 (1/6)		
		P58S	5.4 (2/37)	4.8 (1/21)		11.1 (1/9)	
		Any	35.1 (13/37)	38.1 (8/21)	16.7 (1/6)	33.3 (3/9)	100 (1/1)
3a	NS3	A166S/T	28.6 (2/7)	33.3 (1/3)	33.3 (1/3)	(0/1)	
	NS5A	A30K	14.3 (1/7)	33.3 (1/3)			
		Y93H	14.3 (1/7)		33.3 (1/3)		
		Any	28.6 (2/7)	33.3 (1/3)	33.3 (1/3)	(0/1)	
3b	NS3	Any	(0/4)	(0/1)	(0/2)	(0/1)	
	NS5A	V31M	100 (4/4)	100 (1/1)	100 (2/2)	100 (1/1)	
3k	NS5A	G92E	100 (1/1)	100 (1/1)			

a HCV subtype was determined by phylogenetic analysis of NS3/4A and/or NS5A sequences.

b Polymorphisms relative to the subtype-specific reference sequence at amino acid positions important for the inhibitor class, positions 36, 43, 54, 55, 56, 80, 155, 156, 166 (GT3 only), and 168 in NS3 and positions 24, 28, 30, 31, 32, 58, 92, and 93 in NS5A, at a 15% detection threshold, were included in the analysis.

c GT1- and GT2-infected patients without cirrhosis received 8 weeks of treatment. GT1- and GT2-infected patients with compensated cirrhosis and GT3-infected patients with or without cirrhosis received 12 weeks of treatment.

d IFN-TE, treatment experienced with an interferon-containing regimen but DAA naive; TN, treatment naive.

In studies across overseas regions, among DAA treatment-naive GT1b-infected patients grouped by North America (*n* = 74), Europe (*n* = 266), or ROW (*n* = 126), the prevalences of baseline polymorphisms at position 36, 54, 55, or 56 in NS3 were similar across the 3 regions and Japan, while the prevalence of Q80L was numerically higher in Japan and ROW than in North America and Europe (see Fig. S1 in the supplemental material). The prevalence of baseline polymorphisms in NS5A at position 24, 28, 30, or 93, specifically Q24K, L28M, R30H/Q, or Y93H, was higher in Japan than in any of the other 3 regions (Fig. S1).

Only two HCV GT2 subtypes, GT2a (*n* = 78) and GT2b (*n* = 37), were identified by phylogenetic analysis in CERTAIN-1 and CERTAIN-2 studies; baseline sequences from two of the GT2-infected patients were not available for HCV subtyping by phylogenetic analysis. The majority of GT2-infected patients (67.8%; *n* = 78) were treatment naive without cirrhosis. Baseline polymorphisms in NS3 were detected in 6.3% of the patients across both GT2 subtypes. In GT2a-infected patients, polymorphisms at positions 24, 28, 58, and 92 in NS5A were detected at a low prevalence, while the M31 polymorphism was detected in 92.3% of the patients. In GT2b-infected patients, L28F and the M31 polymorphism in NS5A were detected at prevalences of 16.2% and 83.8%, respectively. L31 in NS5A in GT2b-infected patients was detected at a much higher prevalence in overseas regions (67.9%) than in Japan (10.8%) (Fig. S2).

In GT3a-infected patients (*n* = 7), A166S/T in NS3 was detected in 2 patients, and A30K and Y93H in NS5A were each detected in one patient. Baseline polymorphisms at other amino acid positions important for the PI class were not detected in NS3, and V31M in NS5A was detected in the 4 GT3b-infected patients. The single GT3k-infected patient had G92E in NS5A, while the NS3 gene could not be sequenced due to technical difficulties.

None of the single amino acid polymorphisms observed at baseline in DAA-naive patients that were tested in replicon assays conferred resistance to glecaprevir or pibrentasvir for any genotype (Table 4).

**TABLE 4:** HCV PI and NS5A inhibitor class amino acid positions of interest and resistance profile of glecaprevir and pibrentasvir as determined by a transient-replicon assay

Inhibitor and inhibitor class-specific amino acid positions	HCV GT	Amino acid substitution(s) leading to a change in inhibitor EC_50_ of^*b*^:		
		<10-fold	10- to 100-fold	>100 fold
Glecaprevir, NS3 positions 36, 43, 54, 55, 56, 80, 155, 156, 168^*a*^	1a	V36A/L/M, F43L, T54S, V55I, Y56H, Q80K/L/R, R155K/M/S/T/V, A156G, D168A/E/H/N/V	D168F/Y	A156T
	1b	T54A, V55A, Q80K/L, R155K, A156S, D168A/E/F/H/T/V/Y	D168K	A156T/V
	2a	V55A/I, Y56F/H, D168A/E/H/V/Y	None	A156T/V
	2b	Y56F, D168A/E/F/H/S/T/V/Y	None	A156M/T/V
	3a	R155K, S166T, Q168H	Q80R, Q168L/R	A156G
	4a	R155C, D168V	D168H	A156T/V
	4d	Y56H, D168V	None	None
	5a	None	None	None
	6a	None	D168A/V	D168G/H/Y
Pibrentasvir, NS5A positions 24, 28, 30, 31, 32, 58, 92, 93	1a	K24R, M28A/T/V, Q30E/G/H/K/L/R/Y, L31M/V, P32L, H58C/D/P/R, A92T, Y93C/F/H/L/N/S	Q30D	M28G
	1b	L28M/T, R30Q, L31F/M/V, P58S, A92E/V, Y93H/N/S	None	P32deletion
	2a	T24A/S, F28C/S, K30G/M, M31I, C92S	None	None
	2b	L28F, L31I/M/V, C92S/Y	None	None
	3a	M28T, A30K, Y93H	None	None
	4a	L28I/M/V, L30H, P58L	None	None
	4d	L28V, M31I/L, T58A/P/S	None	None
	5a	L28I, L31F/V	None	None
	6a	L31V, T58A/N	None	None

a Amino acid position 166 in GT3 in NS3 is also considered a position of interest.

b Available data for single amino acid substitutions are included. The fold change was calculated as the ratio of the EC_50_ in a replicon with the amino acid substitution relative to the EC_50_ in the wild-type replicon. Variants that are underlined were detected at baseline in DAA-naive patients.

### Analysis of baseline substitutions in NS3 and NS5A in DAA-experienced patients.

Of the 32 DAA-experienced GT1b-infected patients enrolled, 30 had most recently failed a regimen containing daclatasvir plus asunaprevir (some had failed multiple DAA regimens), and 2 had previous experience with a PI-containing regimen (simeprevir) but not an NS5A inhibitor (see Table S1 in the supplemental material). One additional GT2a-infected patient that had previous experience with the sofosbuvir plus RBV regimen was also enrolled; this patient had T24A and M31 in NS5A at baseline.

**FIG 1:**
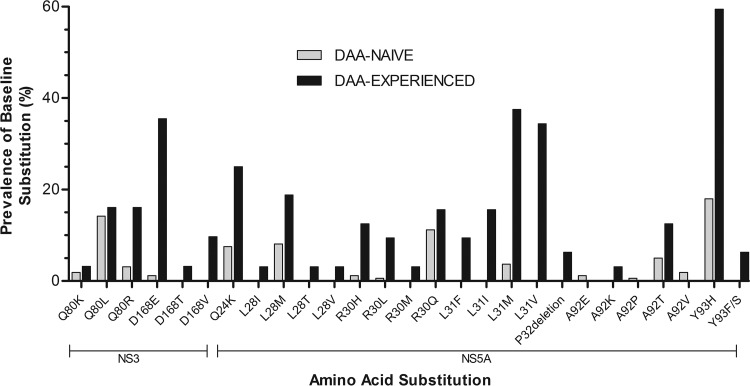
Comparison of prevalences of baseline polymorphisms in NS3 and NS5A in GT1b-infected DAA-naive and DAA-experienced patients. The prevalences of baseline polymorphisms at a 15% detection threshold relative to the GT1b-Con1 reference sequence are shown at amino acid positions at which the overall prevalences between the two patient populations varied.

Baseline substitutions detected in NS3 and NS5A in the GT1b-infected patients are shown in Table 5. A list of baseline substitutions in each patient is shown in Table S1 in the supplemental material. A comparison of specific baseline substitutions in DAA-naive versus DAA-experienced GT1b-infected patients at amino acid positions important for the respective inhibitor class in NS3 and NS5A is shown in Fig. 1. Compared to DAA-naive patients, the prevalences of baseline substitutions at amino acid positions 80 and 168 in NS3 and at amino acid positions 24, 28, 30, 31, 32, 58, 92, and 93 in NS5A were higher in DAA-experienced patients. Specifically, Q80R and D168E/T/V in NS3 and Q24K, L28I/M/T/V, R30H/L/M, L31F/I/M/V, A92K/T, and Y93F/H/S in NS5A were enriched in DAA-experienced patients. The prevalence of D168E/T/V in NS3 was 48.4% (15/31), of which Q80R plus D168E were detected in 12.9% (4/31) of the patients. In NS5A, L31F/I/M/V in combination with Y93H was detected in 59.4% (19/32) of patients. Two or more baseline substitutions at position 24, 28, 30, 31, 32, 58, 92, or 93 in NS5A were detected in 84.4% (27/32) of patients.

**TABLE 5:** Prevalence of baseline substitutions among DAA-experienced GT1b-infected patients

Target	Baseline substitution(s)^*a*^	% of patients with baseline substitution(s) (no. of patients with baseline substitution[s]/total no. of DAA-experienced GT1b-infected patients sequenced)			
		Without cirrhosis		With compensated cirrhosis (PI experienced and NS5A inhibitor experienced)	Total
		PI experienced and NS5A inhibitor naive	PI experienced and NS5A inhibitor experienced		
NS3	Y56F		24.0 (6/25)		19.4 (6/31)
	Q80K/L/R	50.0 (1/2)	32.0 (8/25)	50.0 (2/4)	35.5 (11/31)
	D168E/T/V		52.0 (13/25)	50.0 (2/4)	48.4 (15/31)
	Any	50.0 (1/2)	72.0 (18/25)	75.0 (3/4)	71.0 (22/31)
	Multiple	(0/2)	32.0 (8/25)	25.0 (1/4)	29.0 (9/31)
NS5A	Q24K	50.0 (1/2)	26.9 (7/26)		25.0 (8/32)
	L28I/M/T/V	50.0 (1/2)	26.9 (7/26)		25.0 (8/32)
	R30H/L/M/Q	50.0 (1/2)	38.5 (10/26)		34.4 (11/32)
	L31F/I/M/V		88.5 (23/26)	75.0 (3/4)	81.3 (26/32)
	P32deletion		3.8 (1/26)	25.0 (1/4)	6.3 (2/32)
	P58L	50.0 (1/2)	3.8 (1/26)		6.3 (2/32)
	A92K/T	50.0 (1/2)	15.4 (4/26)		15.6 (5/32)
	Y93F/S		7.7 (2/26)		6.3 (2/32)
	Y93H		61.5 (16/26)	75.0 (3/4)	59.4 (19/32)
	Any	100 (2/2)	96.2 (25/26)	100 (4/4)	96.9 (31/32)
	Multiple	50.0 (1/2)	88.5 (23/26)	75.0 (3/4)	84.4 (27/32)

a Baseline substitutions at amino acid positions important for the inhibitor class, positions 36, 43, 54, 55, 56, 80, 155, 156, and 168 in NS3 and positions 24, 28, 30, 31, 32, 58, 92, and 93 in NS5A, at a 15% detection threshold, were included in the analysis. Multiple indicates patients with 2 or more baseline substitutions.

**FIG 2:**
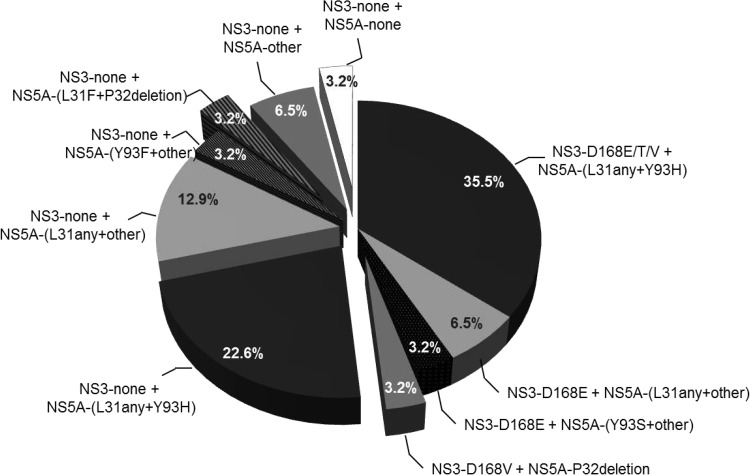
Combinations of baseline substitutions across NS3 and NS5A in GT1b-infected DAA-experienced patients. Shown are the numbers and percentages of patients with baseline substitutions in NS3 (position 168) in combination with NS5A substitutions at position 31, 32, or 93 or at other positions (positions 24, 28, 30, 58, and 92) detected by NGS at a ≥15% prevalence within a patient's viral population.

Baseline substitutions across NS3 (at position 168) and NS5A are summarized in Fig. 2. NS5A-[L31F/I/M/V+Y93H], either in combination with NS3-D168E/T/V or without NS3-D168 substitutions, was detected in 35.5% (11/31) and 22.6% (7/31) of patients, respectively. NS3-D168E in combination with NS5A-L31F/M/V or NS5A-Y93S was detected in 9.7% (3/31) of the patients. One patient had NS3-D168V plus NS5A-P32deletion, and one had NS5A-[L31F+P32deletion] without NS3-D168 substitutions. Seven patients (22.6%) had NS5A substitutions without D168 substitutions in NS3. One patient had no baseline substitutions in NS3 or NS5A.

None of the tested substitutions detected at baseline in NS3 or NS5A, with the exception of the P32deletion in NS5A, conferred resistance to glecaprevir or pibrentasvir *in vitro* in the GT1b replicon assay (Tables 6 and 7).

**TABLE 6:** *In vitro* activity of glecaprevir against amino acid substitutions observed in DAA-experienced HCV GT1b-infected patients

NS3 amino acid substitution(s)^*a*^	Mean glecaprevir EC_50_ ± SD (nM)	Fold change in EC_50_
GT1b wild type	0.47 ± 0.13	
Q80K	0.37 ± 0.03	0.8
Q80L	0.30 ± 0.07	0.6
A156V	839 ± 181	1,786
D168A	0.69 ± 0.11	1.5
D168E	0.40 ± 0.08	0.9
D168T	1.3 ± 0.20	2.8
D168V	1.5 ± 0.43	3.2
A156V + D168V	2,465 ± 1,321	5,244

a Amino acid substitutions that were detected at baseline or at the time of failure in patients experiencing virologic failure are underlined.

**TABLE 7:** *In vitro* activity of pibrentasvir against amino acid substitutions observed in DAA-experienced HCV GT1b-infected patients

NS5A amino acid substitution(s)^*a*^	Mean pibrentasvir EC_50_ ± SD (pM)	Fold change in EC_50_
GT1b wild type	1.9 ± 0.80	
Q24K	3.0 ± 0.59	1.6
L28M	1.8 ± 0.11	1.0
L28T	1.7 ± 0.44	0.9
R30Q	0.88 ± 0.49	0.5
L31F	2.3 ± 0.37	1.2
L31M	2.9 ± 1.2	1.5
L31V	1.5 ± 0.43	0.8
P32deletion	1,968 ± 203	1,036
P58S	2.4 ± 1.3	1.2
A92E	0.92 ± 0.23	0.5
A92V	0.86 ± 0.15	0.5
Y93H	1.1 ± 0.27	0.6
Y93S	0.74 ± 0.24	0.4
Q24K + R30Q	3.0 ± 0.21	1.6
L28M + R30Q	0.79 ± 0.10	0.4
L28M + Y93H	2.2 ± 0.23	1.2
R30Q + Y93H	2.3 ± 0.15	1.2
L31F + P32deletion	38,877 ± 1,227	20,461
L31F + A92E	1.2 ± 0.15	0.6
L31F + Y93H	2.8 ± 0.17	1.5
L31M + Y93H	1.3 ± 0.24	0.7
L31V + A92K	5.0 ± 0.62	2.6
L31V + Y93H	1.7 ± 0.31	0.9
P58S + Y93H	1.5 ± 0.45	0.8
L28M + R30Q + Y93H	1.0 ± 0.24	0.5

a Amino acid substitutions that were detected at baseline or at the time of failure in patients experiencing virologic failure are underlined.

### Impact of baseline polymorphisms/substitutions on treatment outcome.

Among DAA-naive GT1- and GT2-infected patients, there were no virologic failures; therefore, baseline polymorphisms had no impact on treatment outcomes. One GT3b-infected patient and one GT3k-infected patient experienced virologic failure; the GT3b-infected patient had unusually low plasma concentrations of glecaprevir-pibrentasvir (21). Given the low number of GT3-infected patients enrolled in this study (*n* = 12), the impact of baseline polymorphisms on treatment outcomes in GT3-infected patients could not be meaningfully assessed.

Among DAA-experienced GT1b-infected patients, baseline substitutions in NS3 and/or NS5A, including those at amino acid position 168 in NS3 or at position 31 or 93 in NS5A, had no impact on SVR_12_ (see Table S2 in the supplemental material). Two patients with a P32deletion in NS5A experienced virologic failure. This is consistent with the resistance profile of glecaprevir and pibrentasvir, as none of the baseline substitutions, with the exception of the P32deletion in NS5A, conferred resistance to glecaprevir or pibrentasvir (Tables 6 and 7).

### Resistance analysis for patients experiencing virologic failure.

Baseline and postbaseline variants in NS3 and NS5A among the 4 patients experiencing virologic failure are shown in Table 8. Of the 2 GT1b-infected PI- plus NS5A inhibitor-experienced patients with virologic failure, treatment-emergent substitution A156D/V in NS3 was detected in 1 patient (D168V was also present at baseline and postbaseline in this patient). In NS5A, P32deletion (P32L was detected at the 2% but not at the 15% detection threshold) and L31F plus P32deletion were present at both baseline and postbaseline in 1 patient each. D168V plus A156V in NS3 and P32deletion alone or in combination with L31F in NS5A conferred high levels of resistance to glecaprevir and pibrentasvir, respectively. A P32deletion in NS5A also confers high levels of resistance to all the currently marketed NS5A inhibitors, including velpatasvir, ledipasvir, ombitasvir, elbasvir, and daclatasvir (Table 9). Of the 2 GT3-infected patients with virologic failure, treatment-emergent substitutions L28F and/or Y93H was detected in NS5A; NS3 sequences were not available for analysis.

**TABLE 8:** Baseline polymorphisms and treatment-emergent substitutions at DAA class-specific amino acid positions^*c*^

Prior treatment experience	GT	Time of VF	Variant (prevalence in patient's viral population [%])^*a*^			
			NS3		NS5A	
			Baseline	Time of VF	Baseline	Time of VF
PI experienced + NS5A inhibitor experienced	1b	W12		A156D (31.1)	P32L (8.8)	P32L (7.1)
				A156V (66.7)	P32deletion (90.6)	P32deletion (92.6)
			D168V (22.1)	D168V (99.0)		
PI experienced + NS5A inhibitor experienced	1b	PTW4	Y56F (99.2)	Y56F (99.4)	L31F (96.2)	L31F (96.9)
			Q80L (98.5)	Q80L (99.2)	P32deletion (96.4)	P32deletion (97.0)
TN	3k	PTW12	NA	NA		L28F (99.8)
					G92E (99.4)	G92E (99.8)
						Y93H (99.6)
IFN-TE	3b	PTW2	None^*b*^	NA	V31M (99.7)	V31M (99.7)
						Y93H (99.7)

a DAA class-specific polymorphisms/substitutions relative to the subtype-specific reference sequence at the following amino acid positions are listed: positions 36, 54, 55, 56, 80, 155, 156, and 168 in NS3 and positions 24, 28, 29, 30, 31, 32, 54, 58, 62, 92, and 93 in NS5A for GT1b and positions 36, 43, 54, 55, 56, 80, 155, 156, 166, and 168 in NS3 and positions 24, 28, 29, 30, 31, 32, 58, 92, and 93 in NS5A for GT3. The prevalences of variants within a patient's viral population are also listed.

b “None” indicates that DAA class-specific polymorphisms/substitutions were not detected.

c GT, HCV subtype determined by phylogenetic analysis; IFN-TE, treatment experienced with an interferon-containing regimen but DAA naive; NA, not available due to technical reasons; PTW, posttreatment week; TN, treatment naive; VF, virologic failure; W12, week 12.

**TABLE 9:** *In vitro* activities of NS5A inhibitors against NS5A P32deletion with or without additional NS5A substitutions in the HCV GT1b-Con1 replicon

NS5A inhibitor	Mean EC_50_ ± SD (pM) for wild-type GT1b	P32deletion		L31F + P32deletion	
		Mean EC_50_ ± SD (pM)	Fold change in EC_50_	Mean EC_50_ ± SD (pM)	Fold change in EC_50_
Pibrentasvir	1.9 ± 0.8	1,968 ± 203	1,036	38,877 ± 1,227	20,461
Ombitasvir	0.79 ± 0.25	1,363,857 ± 347,809	1,726,401	1,076,900 ± 214,654	1,363,165
Daclatasvir	10.6 ± 2.0	3,497,200 ± 1,004,375	329,925	4,127,000 ± 224,301	389,340
Elbasvir	3.2 ± 1.4	368,900 ± 176,726	115,281	321,900 ± 61,946	100,594
Ledipasvir	1.6 ± 0.62	845,233 ± 104,697	528,271	723,967 ± 54,768	452,479
Velpatasvir	4.8 ± 0.77	196,200 ± 30,448	40,875	302,133 ± 20,880	62,944

## DISCUSSION

The efficacy and safety of the once-daily (QD), RBV-free glecaprevir-pibrentasvir regimen were evaluated in HCV-infected patients in 2 phase 3 studies in Japan, CERTAIN-1 and CERTAIN-2 (19–21). Consistent with the epidemiology of the HCV subtype distribution in Japan, 7 subtypes, including 170 GT1-infected (4 GT1a and 166 GT1b), 117 GT2-infected (78 GT2a, 37 GT2b, and 2 GT2 with an undetermined subtype), and 12 GT3-infected (7 GT3a, 4 GT3b, and 1 GT3k) DAA-naive patients and 32 GT1b- and 1 GT2a-infected DAA-experienced patients were enrolled. There were no virologic failures among DAA-naive GT1- or GT2-infected noncirrhotic patients treated for 8 weeks or DAA-naive patients with compensated cirrhosis treated for 12 weeks. Among 33 DAA-experienced patients (30 were experienced to daclatasvir plus asunaprevir regimen) treated for 12 weeks, 2 experienced virologic failure. Two of 12 GT3-infected patients treated for 12 weeks experienced virologic failure. The glecaprevir-pibrentasvir regimen at the above-listed durations for the various patient populations recently received marketing approval in Japan.

Baseline polymorphisms at amino acid positions important for the PI class (position 155, 156, or 168) were rarely detected. In NS5A, the baseline prevalence of Q24K (8%), L28M (8%), R30Q (11%), or Y93H (18%) was higher in Japanese GT1b-infected patients than in patients from other geographic regions. The NS5A Y93H polymorphism in GT1b confers resistance to NS5A inhibitors ombitasvir (77-fold), daclatasvir (24-fold), elbasvir (17-fold), and ledipasvir (1,807-fold) (22–26). According to JSH guidelines for the management of hepatitis C virus infection, ombitasvir-paritaprevir-ritonavir or daclatasvir plus asunaprevir is not recommended for use in GT1b-infected patients with a baseline NS5A Y93H polymorphism (10). In Japanese clinical studies with elbasvir plus grazoprevir in GT1b-infected patients for 12 weeks, SVR_12_ rates in patients with and without baseline NS5A polymorphisms were 93.1% (54/58) and 98.9% (260/263), respectively, and the SVR_12_ rate in patients with Y93 variants was 93% (40/43) (11). Additionally, a recent real-life multiple-cohort study in Japan showed a similar negative impact of these baseline polymorphisms on GT1b-infected patients with cirrhosis (treatment naive, prior IFN/RBV failure, or prior PI failure) receiving sofosbuvir-ledipasvir; baseline NS5A polymorphisms at amino acid positions 31 and/or 93 were associated with a reduced SVR_12_ rate of 90.7% (49/54), compared to an SVR_12_ rate of 99.4% (154/155) in patients without baseline polymorphisms (27). Thus, in Japanese GT1b-infected patients, the preexistence of NS5A polymorphisms, especially Y93H, had a negative impact on SVR_12_ rates in patients receiving regimens containing any of the these NS5A inhibitors. Pibrentasvir is a next-generation NS5A inhibitor that retains its activity against Y93H in NS5A in GT1 replicons (14). Consistent with the high barrier to resistance *in vitro*, baseline polymorphisms in NS5A had no impact on treatment outcome with the glecaprevir-pibrentasvir regimen, and there were no virologic failures among DAA-naive GT1-infected patients without cirrhosis (8-week treatment duration) or with compensated cirrhosis (12-week treatment duration) in the CERTAIN-1 study. Very few HCV-infected patients (<1% of GT1-infected patients) are infected with GT1a in Japan (8), and consistent with the epidemiology in Japan, only 4 DAA-naive GT1a-infected patients were enrolled in CERTAIN-1. Although the data set for the analysis of baseline polymorphisms in GT1a-infected patients was limited in this study, 2 of the 4 patients had M28V, Q30H, or Y93F in NS5A at baseline, and all 4 achieved SVR_12_. In global studies with the glecaprevir-pibrentasvir regimen, baseline polymorphisms in NS3 at amino acid position 155, 156, or 168 in NS3 were detected in 2.1% (8/384) of DAA treatment-naive GT1a-infected patients, and those in NS5A at amino acid position 24, 28, 30, 31, 58, 92, or 93 in NS5A were detected in 20.5% (78/380) of DAA treatment-naive GT1a-infected patients (16). Baseline polymorphisms had no impact on treatment outcomes in GT1a-infected patients irrespective of the treatment duration or cirrhosis status (16, 17).

The NS5A M31 polymorphism was predominant in patients infected with GT2a (92.3%) and GT2b (83.8%) in Japan, while in other geographic regions, M31 was predominant in GT2a-infected patients but was detected in only 31.6% of the GT2b-infected patients. First-generation DAA regimens that were effective against GT1 in Japan have been less successful in treating GT2-infected patients because the NS5A M31 polymorphism reduces susceptibility to first-generation NS5A inhibitors such as daclatasvir (>1,000-fold) or ledipasvir (12-fold) and reduces the barrier to resistance for ombitasvir (23, 25, 28). Glecaprevir and pibrentasvir have potent activity against both GT2a and GT2b replicons irrespective of the presence of common polymorphisms and have a higher barrier to resistance than the first-generation PIs or NS5A inhibitors (14, 15). Consistent with their respective *in vitro* profiles, baseline polymorphisms in NS3 or NS5A had no impact on treatment outcomes with the glecaprevir-pibrentasvir regimen, and there were no virologic failures among DAA-naive GT2-infected patients without cirrhosis or with compensated cirrhosis in the CERTAIN-1 and CERTAIN-2 studies.

The incidence of HCV GT3 is low in Japan, and the only approved DAA-based treatment option (prior to the approval of glecaprevir-pibrentasvir) included sofosbuvir plus RBV for 24 weeks, which had an SVR rate of 85% (29). In the CERTAIN-1 study, the SVR_12_ rate in GT3-infected patients was 83.3% (10/12) with the 12-week glecaprevir-pibrentasvir regimen; 1 of 4 GT3b-infected and 1 of 1 GT3k-infected patients experienced virologic failure, and all 7 GT3a-infected patients achieved SVR_12_. In global studies with glecaprevir-pibrentasvir, the resistance analysis data set included data for 627 GT3a-, 6 GT3b-, and 2 GT3i-infected subjects. Overall, baseline NS3 and most of the NS5A polymorphisms had no impact on the SVR_12_ rate; A30K in NS5A was associated with lower SVR_12_ rates in GT3a-infected pegIFN/RBV treatment-experienced noncirrhotic patients receiving 12 weeks of treatment (16). In the CERTAIN-1 study, A30K was detected in 1 GT3a-infected subject; however, K30 in NS5A is present in the GT3b and GT3k reference sequences and thus was not counted as a polymorphism. The overall prevalence of K30 in NS5A among the 12 GT3-infected patients in CERTAIN-1 was 50% (6/12), and 2 of these patients experienced virologic failure. Data on the *in vitro* activity of pibrentasvir against GT3b and GT3k replicons containing K30 with or without the treatment-emergent Y93H substitution are not available. Additionally, the GT3b-infected patient experiencing virologic failure had received prior IFN-based treatment, and the plasma concentrations were substantially low for both glecaprevir and pibrentasvir during weekly treatment visits for this patient (21). Therefore, it is unclear how much of an impact, if any, K30 in NS5A had on treatment outcomes in the GT3b- and GT3k-infected patients experiencing virologic failure.

For GT1-infected patients who had failed previous PI-NS5A inhibitor therapy, sofosbuvir-ledipasvir was the only IFN-free DAA regimen recommended in the 2016 JSH guidelines for the management of hepatitis C virus infection and only if the virus did not harbor multiple polymorphisms at positions 31 plus 93 in NS5A (10). Due to insufficient evidence on the impact of DAA resistance in sofosbuvir-ledipasvir failures on future treatment, the 2016 JSH guidelines also considered waiting for new drug development as an option. In 2014, the daclatasvir plus asunaprevir regimen was the first IFN-free regimen approved in Japan, leading to the significant usage of this regimen prior to the approval of subsequent DAA regimens. As baseline L31 and/or Y93 polymorphisms had a negative impact on treatment outcome in patients administered daclatasvir plus asunaprevir, the overall the SVR_12_ rate in a phase 3 clinical study was only 84.7% in GT1-infected patients (30). In addition, patients experiencing virologic failure were left with D168 substitutions in NS3 and L31 and/or Y93 substitutions in NS5A (30). A recent real-life study of daclatasvir plus asunaprevir-experienced patients retreated with sofosbuvir-ledipasvir demonstrated a high virologic failure rate of around 36% (31–33). Recent approval of the glecaprevir-pibrentasvir regimen in Japan will help address retreatment options for prior DAA-experienced patients, including those with experience with an NS5A inhibitor alone or a PI plus an NS5A inhibitor.

The majority of the DAA-experienced GT1b-infected patients enrolled in CERTAIN-1 (30/32) most recently had experience with a daclatasvir plus asunaprevir-containing regimen, and 2/32 patients had experience with a simeprevir plus IFN-containing regimen. One GT2a-infected patient had experience with sofosbuvir plus RBV. In daclatasvir plus asunaprevir-experienced patients, baseline Q80R and D168E/T/V substitutions in NS3 and Q24K, L28I/M/T/V, R30H/L/M, L31F/I/M/V, A92K/T, and Y93F/H/S substitutions in NS5A were enriched relative to the prevalence observed in DAA-naive patients in the study. Overall, 97% (32/33) of patients had baseline substitutions in NS3 and/or NS5A. D168E/T/V (15/30 patients) in NS3 and L31F/I/M/V (26/30) and Y93F/H/S (21/30) in NS5A were the most common baseline substitutions detected in previously daclatasvir plus asunaprevir-experienced patients. D168E/T/V in NS3 was detected in combination with L31F/I/M/V and/or Y93F/H/S in NS5A in 15 patients; all 19 patients with NS5A Y93H also had L31 substitutions. Baseline substitutions observed in these DAA-experienced patients are representative of substitutions commonly selected in patients experiencing virologic failure with daclatasvir plus asunaprevir with or without beclabuvir; ombitasvir-paritaprevir-ritonavir; sofosbuvir-ledipasvir; sofosbuvir-velpatasvir; or elbasvir plus grazoprevir (30, 34–37). These amino acid substitutions, alone or in combination, remained susceptible to glecaprevir or pibrentasvir in *in vitro* replicon assays (14, 15). Consistent with this high barrier to resistance, the common and prevalent baseline substitutions in NS3 and/or NS5A had no impact on treatment outcomes. The only 2 patients experiencing virologic failure both had a P32deletion in NS5A, which had a baseline prevalence of 6.7% (2/30) in daclatasvir plus asunaprevir-experienced patients in this study. A P32deletion in NS5A has been observed as an uncommon treatment-emergent substitution in patients who have failed treatment with a daclatasvir-containing regimen (38–40) and confers >1,000-fold resistance to pibrentasvir, accounting for the negative impact of this substitution on treatment outcome. The P32deletion also confers >10,000-fold resistance to all of the other currently marketed NS5A inhibitors, daclatasvir, ledipasvir, velpatasvir, elbasvir, or ombitasvir (Table 9).

In summary, the CERTAIN-1 and CERTAIN-2 studies in Japan evaluated a glecaprevir-pibrentasvir regimen in 332 treatment-naive, IFN-experienced, and DAA-experienced patients, including those without cirrhosis and with compensated cirrhosis and those with and without severe renal impairment. A high overall SVR_12_ rate of 98.3% was observed across DAA-naive patients treated with glecaprevir-pibrentasvir. In addition, a high SVR_12_ rate of 93.9% was observed in patients with previous DAA experience. In both DAA-naive and DAA-experienced patients, the treatment outcome was not affected by baseline substitutions in NS3 and/or NS5A that are commonly selected by currently available PIs and/or NS5A inhibitors, including those at amino acid positions 168 in NS3 and 31 or 93 in NS5A. Unlike the first-generation HCV pegIFN/RBV-free DAA regimens, the glecaprevir-pibrentasvir regimen allows a simplified treatment algorithm without the need for baseline resistance testing or HCV subtyping in DAA-naive GT1- and GT2-infected patients in Japan.

## MATERIALS AND METHODS

### Study design.

CERTAIN-1 and CERTAIN-2 study designs, randomization procedures, and efficacy and safety analyses were previously described (19–21). Briefly, CERTAIN-1 (ClinicalTrials.gov identifier NCT02707952) is a phase 3, open-label, multicenter study assessing the safety and efficacy of glecaprevir-pibrentasvir (300 mg/120 mg) once daily in Japanese patients with HCV infection. The study was composed of two substudies: in substudy 1, DAA-naive patients with GT1 HCV infection without cirrhosis and without the Y93H polymorphism were randomized to 8 weeks of treatment with glecaprevir-pibrentasvir (arm A) or 12 weeks of treatment with ombitasvir-paritaprevir-ritonavir (arm B). Patients without the NS5A Y93H polymorphism were randomized 2:1 to arm A or B, while patients with the Y93H polymorphism were assigned to arm A only. HCV NS5A population sequencing was performed by a central laboratory at the screening visit for substudy 1 patients to detect the presence or absence of the Y93H polymorphism (approximately a 15% detection threshold for Y93H). In substudy 2, DAA-naive patients with GT1 HCV infection and with compensated cirrhosis were assigned to 12 weeks of glecaprevir-pibrentasvir (300 mg/120 mg) once daily. The remainder of the patients in substudy 2 belonged to four special populations: (i) GT1- or GT2-infected patients who failed prior DAA treatment, including patients with compensated cirrhosis; (ii) GT1- or GT2-infected patients with severe renal impairment and compensated cirrhosis; (iii) GT3-infected patients who were treatment naive or IFN treatment experienced; and (iv) GT1- or GT2-infected patients with severe renal impairment without cirrhosis. The first 3 cohorts received glecaprevir-pibrentasvir (300 mg/120 mg) for 12 weeks, and the fourth cohort received treatment for 8 weeks. Although substudy 2 of CERTAIN-1 was open to patients infected with GT1 to GT6, no subjects with GT4, GT5, or GT6 infection enrolled, due to the very low prevalence of these genotypes in Japan. Patients with severe renal impairment included patients with an estimated glomerular filtration rate (eGFR) of <30 ml/min/1.73 m^2^ (using the MDRD method modified for Japanese population, where eGFR_J_ = 194 × serum creatinine^−1.094^ × age^−0.287^ × 0.739 [if female]) and patients with end-stage renal disease requiring treatment with chronic intermittent hemodialysis. Patients enrolled in CERTAIN-2 (ClinicalTrials.gov identifier NCT02723084) had GT2 infection without cirrhosis and were randomized 2:1 to 8 weeks of treatment with glecaprevir-pibrentasvir (arm A) or 12 weeks of treatment with SOF (400 mg QD) plus RBV (600 to 1,000 mg, weight based, divided twice a day [BID]) (arm B). Patients in arm B of CERTAIN-1 and CERTAIN-2 were excluded from the resistance analyses.

Available samples obtained from GT1b-, GT2a-, and GT2b-infected patients enrolled in North America (Canada, Mexico, Puerto Rico, and the United States), Europe (Austria, Belgium, France, Germany, Greece, Hungary, Italy, Lithuania, Poland, Portugal, Romania, Spain, Sweden, Switzerland, and the United Kingdom), or ROW (Australia, Chile, Israel, South Korea, New Zealand, South Africa, and Taiwan) in the clinical studies SURVEYOR-II, ENDURANCE-1, ENDURANCE-2, EXPEDITION-1, and EXPEDITION-4 were utilized for baseline sequence analysis comparisons (16, 41–43).

All patients provided written, informed consent to participate, and the study was conducted in accordance with the ethical guidelines of the Declaration of Helsinki and the International Conference on Harmonization good clinical practice guidelines. The study was approved by the institutional review board of each study site prior to the initiation of any screening or study-specific procedures.

### Sample processing.

The Versant HCV Genotype Inno-LiPA assay v2.0 (LiPA 2.0), conducted by the Central Lab, was used to determine HCV genotypes for the enrollment of patients into clinical studies. However, for non-HCV GT1, the LiPA 2.0 assay is unable to accurately identify the viral subtype. The viral subtype was therefore determined by phylogenetic analysis of a 329-nucleotide region of HCV NS5B (44, 45). Results from the NS5B phylogenetic analysis were used to assign a preliminary subtype to each HCV-infected patient sample, which then determined the subtype-specific reverse transcriptase PCR (RT-PCR) and nested PCR primer sets used for the independent amplification of NS3/4A and NS5A genes from baseline samples. Subtype-specific RT-PCR, nested PCR, and sequencing primers were designed based on the alignments of available sequences in GenBank and the European HCV database. The primers were designed in conserved regions specific to the gene of interest, and nucleotide degeneracies were incorporated in positions where significant variability existed among the sequences. Only samples with ≥1,000 IU/ml HCV RNA were amplified in order to reduce the chances of oversampling bias. For samples with ≤50,000 IU/ml HCV RNA, RT-PCRs were done in triplicate, and the products were pooled prior to their use as a template for nested PCR. Nested PCR products encompassing the genes encoding full-length NS3/4A or NS5A were analyzed by next-generation sequencing (NGS) analysis performed by the DDL Diagnostic Laboratory (Rijswijk, Netherlands).

### NGS analysis parameters.

PCR amplicons were purified by using AMPure XP beads (Beckman Coulter Genomics, Danvers, MA) and quantified by using the Quant-iT PicoGreen double-stranded DNA (dsDNA) kit (Life Technologies, Carlsbad, CA). The DNA was then fragmented and tagged by using the Nextera XT sample preparation kit (Illumina, San Diego, CA) according to the manufacturer's instructions. Index primers were added by limited-cycle PCR using the Nextera XT Index kit (Illumina, San Diego, CA), and samples were normalized by using beads with maximum binding capacity according to instructions provided with the Nextera XT sample preparation kit. Multiplexed paired-end sequencing was conducted on the Illumina MiSeq platform using the MiSeq v2 sequencing kit with 300 cycles (Illumina, San Diego, CA). Demultiplexed FASTQ files were then mapped against the reference sequence by using CLC Genomics Workbench software (CLCbio, Denmark). Sequences were trimmed to remove nucleotides with a quality score of <30. The attempted minimum coverage is 5,000 sequencing reads per nucleotide position. An amino acid variant report relative to a subtype-specific reference sequence was generated with Athena pipeline proprietary software. The threshold for the detection of nucleotide polymorphisms by NGS was set at 2%.

### HCV genotype and subtype classification.

For each sample analyzed by NGS, a consensus sequence was generated for each target gene from the NGS nucleotide sequences, with an ambiguity setting of 0.25. Phylogenetic analyses were subsequently conducted by using the available full-length HCV NS3/4A and NS5A consensus nucleotide sequences in order to confirm the subtype assignment. Nucleotide sequences for NS3/4A and NS5A were aligned by using the MAFFT sequence alignment method (46). Phylogenetic trees were constructed by using the neighbor-joining tree-building method with the HKY85 nucleotide substitution model (47, 48). The reliability of the tree topology was examined by using bootstrap analysis, and 1,000 bootstrapping replicates were utilized to generate a consensus tree with a 50% threshold cutoff. Nucleotide alignments and phylogenetic trees were generated by using Geneious software (Biomatters Ltd., Auckland, New Zealand). The final HCV subtype assignment was determined by consensus between NS3/4A and NS5A phylogenetic analysis results. If sequences were not available for phylogenetic analyses, subtype assignment by the LiPA 2.0 assay was utilized.

### Resistance analyses of data from CERTAIN-1 and CERTAIN-2.

Data were grouped by HCV subtype, treatment duration, prior HCV treatment experience, or cirrhosis status and broadly included the following analyses: (i) prevalence of polymorphisms at baseline at amino acid positions important for the NS3 protease and NS5A inhibitor classes at a 15% NGS detection threshold relative to the subtype-specific reference sequence for GT1a-H77 (GenBank accession number NC_004102), GT1b-Con1 (GenBank accession number AJ238799), GT2a-JFH-1 (GenBank accession number AB047639), GT2b-HC-J8 (GenBank accession number D10988), GT3a-S52 (GenBank accession number GU814263), GT3b-HCV-Tr (GenBank accession number D49374), or GT3k-JK049 (GenBank accession number D63821); (i) impact of baseline polymorphisms on the treatment response (SVR_12_); and (iii) analysis of baseline polymorphisms and treatment-emergent substitutions in patients experiencing virologic failure (at a 2% detection threshold). The detection threshold of 15% for the analysis of baseline polymorphisms was chosen because lower detection thresholds will likely increase the reported prevalences of baseline polymorphisms, leading to an underestimation of the impact of polymorphisms on treatment outcome. For patients experiencing virologic failure, it is important to understand whether a substitution is enriched at the time of failure relative to its prevalence at baseline as a result of drug pressure; therefore, a more conservative detection threshold of 2% was utilized to ensure that any enrichment was captured. In DAA-naive patients, polymorphism was defined as a baseline amino acid difference relative to the appropriate subtype-specific reference sequence. In DAA-experienced patients, a variant(s) relative to the appropriate subtype-specific reference sequence could have resulted from a prior treatment regimen and is therefore referred to as a baseline substitution(s).

### Antiviral activity against a panel of NS3 or NS5A variants.

The methods for assessing the effects of individual amino acid substitutions on the activity of an inhibitor in HCV replicon cell culture assays were described previously (14, 15). NS3 and NS5A substitutions were each introduced into the subtype-specific subgenomic replicon plasmid by using the Change-IT Multiple Mutation site-directed mutagenesis kit (Affymetrix, Santa Clara, CA), or synthetic DNA constructs encoding NS3 and NS5A substitutions (Integrated DNA Technologies, Coralville, IA) were inserted into a subtype-specific subgenomic replicon plasmid. In a transient assay, the replicon RNA containing the substitutions was transfected via electroporation into a Huh7 cell line. Glecaprevir, pibrentasvir, ombitasvir, and daclatasvir were synthesized at AbbVie. Elbasvir was purchased from ACME Bioscience (Palo Alto, CA), ledipasvir was purchased from Cayman Chemical (Ann Arbor, MI), and velpatasvir was purchased from eNovation Chemicals (Bridgewater, NJ). The luciferase activity in the cells was measured by using an EnVision multilabel plate reader (Perkin-Elmer, Waltham, MA). The 50% effective concentrations (EC_50_s) were calculated by using nonlinear regression curve fitting to the 4-parameter logistic equation in Prism5 software (GraphPad Software Inc., La Jolla, CA). Mean EC_50_s and standard deviations were calculated from at least 3 independent experiments.

## Supplementary Material

Supplemental material

## References

[1] World Health Organization. 2017 Global hepatitis report 2017. World Health Organization, Geneva, Switzerland.

[2] Chung H, Ueda T, Kudo M 2010 Changing trends in hepatitis C infection over the past 50 years in Japan. Intervirology 53:39–43. doi:10.1159/000252782. 20068339

[3] Smith DB, Bukh J, Kuiken C, Muerhoff AS, Rice CM, Stapleton JT, Simmonds P 2014 Expanded classification of hepatitis C virus into 7 genotypes and 67 subtypes: updated criteria and genotype assignment Web resource. Hepatology 59:318–327. doi:10.1002/hep.26744. 24115039 PMC4063340

[4] Hayashi K, Fukuda Y, Nakano I, Katano Y, Toyoda H, Yokozaki S, Hayakawa T, Morita K, Nishimura D, Kato K, Urano F, Takamatsu J 2003 Prevalence and characterization of hepatitis C virus genotype 4 in Japanese hepatitis C carriers. Hepatol Res 25:409–414. doi:10.1016/S1386-6346(03)00016-0. 12699851

[5] Kobayashi M, Chayama K, Arase Y, Tsubota A, Saitoh S, Suzuki Y, Ikeda K, Matsuda M, Koike H, Hashimoto M, Kumada H 1999 Enzyme-linked immunosorbent assay to detect hepatitis C virus serological groups 1 to 6. J Gastroenterol 34:505–509. doi:10.1007/s005350050304. 10452685

[6] Kobayashi M, Kumada H, Chayama K, Arase Y, Saitou S, Tsubota A, Koida I, Ikeda K, Hashimoto M, Iwasaki S 1994 Prevalence of HCV genotype among patients with chronic liver diseases in the Tokyo metropolitan area. J Gastroenterol 29:583–587. doi:10.1007/BF02365439. 8000505

[7] Toyoda H, Kumada T, Takaguchi K, Shimada N, Tanaka J 2014 Changes in hepatitis C virus genotype distribution in Japan. Epidemiol Infect 142:2624–2628. doi:10.1017/S0950268814000478. 24598252 PMC9151254

[8] Takada A, Tsutsumi M, Okanoue T, Matsushima T, Komatsu M, Fujiyama S 1996 Distribution of the different subtypes of hepatitis C virus in Japan and the effects of interferon: a nationwide survey. J Gastroenterol Hepatol 11:201–207. doi:10.1111/j.1440-1746.1996.tb00063.x. 8742914

[9] Toyota J, Karino Y, Suzuki F, Ikeda F, Ido A, Tanaka K, Takaguchi K, Naganuma A, Tomita E, Chayama K, Fujiyama S, Inada Y, Yoshiji H, Watanabe H, Ishikawa H, Hu W, McPhee F, Linaberry M, Yin PD, Swenson ES, Kumada H 2017 Daclatasvir/asunaprevir/beclabuvir fixed-dose combination in Japanese patients with HCV genotype 1 infection. J Gastroenterol 52:385–395. doi:10.1007/s00535-016-1245-6. 27502287

[10] Asahina Y, Izumi N, Hiromitsu K, Kurosaki M, Koike K, Suzuki F, Takikawa H, Tanaka A, Tanaka E, Tanaka Y, Tsubouchi H, Hayashi N, Hiramatsu N, Yotsuyanagi H 2016 JSH guidelines for the management of hepatitis C virus infection: a 2016 update for genotype 1 and 2. Hepatol Res 46:129–165. doi:10.1111/hepr.12645. 26876581

[11] Kumada H, Suzuki Y, Karino Y, Chayama K, Kawada N, Okanoue T, Itoh Y, Mochida S, Toyoda H, Yoshiji H, Takaki S, Yatsuzuka N, Yodoya E, Iwasa T, Fujimoto G, Robertson MN, Black S, Caro L, Wahl J 2017 The combination of elbasvir and grazoprevir for the treatment of chronic HCV infection in Japanese patients: a randomized phase II/III study. J Gastroenterol 52:520–533. doi:10.1007/s00535-016-1285-y. 27873094 PMC5357479

[12] Omata M, Nishiguchi S, Ueno Y, Mochizuki H, Izumi N, Ikeda F, Toyoda H, Yokosuka O, Nirei K, Genda T, Umemura T, Takehara T, Sakamoto N, Nishigaki Y, Nakane K, Toda N, Ide T, Yanase M, Hino K, Gao B, Garrison KL, Dvory-Sobol H, Ishizaki A, Omote M, Brainard D, Knox S, Symonds WT, McHutchison JG, Yatsuhashi H, Mizokami M 2014 Sofosbuvir plus ribavirin in Japanese patients with chronic genotype 2 HCV infection: an open-label, phase 3 trial. J Viral Hepat 21:762–768. doi:10.1111/jvh.12312. 25196837

[13] Sato K, Chayama K, Alves K, Toyoda H, Suzuki F, Kato K, Rodrigues L Jr, Zhang X, Setze C, Pilot-Matias T, Burroughs M, Redman R, Kumada H 2017 Randomized phase 3 trial of ombitasvir/paritaprevir/ritonavir and ribavirin for hepatitis C virus genotype 2-infected Japanese patients. Adv Ther 34:1449–1465. doi:10.1007/s12325-017-0506-y. 28536999

[14] Ng TI, Krishnan P, Pilot-Matias T, Kati W, Schnell G, Beyer J, Reisch T, Lu L, Dekhtyar T, Irvin M, Tripathi R, Maring C, Randolph JT, Wagner R, Collins C 2017 In vitro antiviral activity and resistance profile of the next-generation hepatitis C virus NS5A inhibitor pibrentasvir. Antimicrob Agents Chemother 61:e02558-16. doi:10.1128/AAC.02558-16. 28193664 PMC5404558

[15] Ng T, Tripathi R, Reisch T, Lu L, Middleton T, Hopkins T, Pithawalla R, Irvin M, Dekhtyar T, Krishnan P, Schnell G, Beyer J, McDaniel K, Ma J, Wang G, Jiang L-J, Or YS, Kempf D, Pilot-Matias T, Collins C 30 10 2017 In vitro antiviral activity and resistance profile of the next-generation hepatitis C virus NS3/4A protease inhibitor glecaprevir. Antimicrob Agents Chemother doi:10.1128/AAC.01620-17. PMC5740381 29084747

[16] Krishnan P, Schnell G, Tripathi R, Ng T, Reisch T, Beyer J, Dekhtyar T, Irvin M, Xie W, Larsen L, Mensa F, Pilot-Matias T, Collins C 2017 Pooled resistance analysis in HCV genotype 1-6-infected patients treated with glecaprevir/pibrentasvir in phase 2 and 3 clinical trials. J Hepatol 66(Suppl 1):S500. doi:10.1016/S0168-8278(17)31399-5. PMC6153825 30061289

[17] AbbVie Inc. 2017 Mavyret (glecaprevir and pibrentasvir) package insert. AbbVie Inc, North Chicago, IL.

[18] AbbVie Inc. 2017 Maviret (glecaprevir and pibrentasvir) summary of product characteristics. AbbVie Inc, North Chicago, IL.

[19] Toyoda H, Chayama K, Suzuki F, Sato K, Atarashi T, Watanabe T, Atsukawa M, Naganuma A, Notsumata K, Osaki Y, Nakamuta M, Takaguchi K, Saito S, Kato K, Pugatch D, Burroughs M, Redman R, Alves K, Pilot-Matias TJ, Oberoi RK, Fu B, Kumada H 2 9 2017 Efficacy and safety of glecaprevir/pibrentasvir in Japanese patients with chronic genotype 2 hepatitis C virus infection. Hepatology doi:10.1002/hep.29510. PMC5814891 28865152

[20] Chayama K, Suzuki F, Karino Y, Kawakami Y, Sato K, Atarashi T, Naganuma A, Watanabe T, Eguchi Y, Yoshiji H, Seike M, Takei Y, Kato K, Alves K, Burroughs M, Redman R, Pugatch DL, Pilot-Matias TJ, Krishnan P, Oberoi RK, Xie W, Kumada H 25 9 2017 Efficacy and safety of glecaprevir/pibrentasvir in Japanese patients with chronic genotype 1 hepatitis C virus infection with and without cirrhosis. J Gastroenterol doi:10.1007/s00535-017-1391-5. PMC5866824 28948366

[21] Kumada H, Watanabe T, Suzuki F, Ikeda K, Sato K, Toyoda H, Atsukawa M, Ido A, Takaki A, Enomoto N, Kato K, Alves K, Burroughs M, Redman R, Pugatch D, Pilot-Matias T, Krishnan P, Oberoi R, Xie W, Chayama K 20 10 2017 Efficacy and safety of glecaprevir/pibrentasvir in HCV-infected Japanese patients with prior DAA experience, severe renal impairment or genotype 3 infection. J Gastroenterol doi:10.1007/s00535-017-1396-0. PMC5866827 29052790

[22] Krishnan P, Beyer J, Mistry N, Koev G, Reisch T, DeGoey D, Kati W, Campbell A, Williams L, Xie W, Setze C, Molla A, Collins C, Pilot-Matias T 2015 In vitro and in vivo antiviral activity and resistance profile of ombitasvir, an inhibitor of hepatitis C virus NS5A. Antimicrob Agents Chemother 59:979–987. doi:10.1128/AAC.04226-14. 25451055 PMC4335823

[23] Wang C, Jia L, O'Boyle DR II, Sun JH, Rigat K, Valera L, Nower P, Huang X, Kienzle B, Roberts S, Gao M, Fridell RA 2014 Comparison of daclatasvir resistance barriers on NS5A from hepatitis C virus genotypes 1 to 6: implications for cross-genotype activity. Antimicrob Agents Chemother 58:5155–5163. doi:10.1128/AAC.02788-14. 24936600 PMC4135806

[24] Liu R, Curry S, McMonagle P, Yeh WW, Ludmerer SW, Jumes PA, Marshall WL, Kong S, Ingravallo P, Black S, Pak I, DiNubile MJ, Howe AY 2015 Susceptibilities of genotype 1a, 1b, and 3 hepatitis C virus variants to the NS5A inhibitor elbasvir. Antimicrob Agents Chemother 59:6922–6929. doi:10.1128/AAC.01390-15. 26303801 PMC4604396

[25] Cheng G, Tian Y, Doehle B, Peng B, Corsa A, Lee YJ, Gong R, Yu M, Han B, Xu S, Dvory-Sobol H, Perron M, Xu Y, Mo H, Pagratis N, Link JO, Delaney W 2016 In vitro antiviral activity and resistance profile characterization of the hepatitis C virus NS5A inhibitor ledipasvir. Antimicrob Agents Chemother 60:1847–1853. doi:10.1128/AAC.02524-15. 26824950 PMC4775926

[26] Wyles D, Mangia A, Cheng W, Shafran S, Schwabe C, Ouyang W, Hedskog C, McNally J, Brainard DM, Doehle BP, Svarovskaia E, Miller MD, Mo H, Dvory-Sobol H 26 6 2017 Long-term persistence of HCV NS5A resistance associated substitutions after treatment with the HCV NS5A inhibitor, ledipasvir, without sofosbuvir. Antivir Ther doi:10.3851/IMP3181. 28650844

[27] Ogawa E, Furusyo N, Nomura H, Dohmen K, Higashi N, Takahashi K, Kawano A, Azuma K, Satoh T, Nakamuta M, Koyanagi T, Kato M, Shimoda S, Kajiwara E, Hayashi J 2017 NS5A resistance-associated variants undermine the effectiveness of ledipasvir and sofosbuvir for cirrhotic patients infected with HCV genotype 1b. J Gastroenterol 52:845–854. doi:10.1007/s00535-016-1290-1. 27913920

[28] Schnell G, Tripathi R, Krishnan P, Beyer J, Reisch T, Irvin M, Dekhtyar T, Setze C, Rodrigues L Jr, Alves K, Burroughs M, Redman R, Chayama K, Kumada H, Collins C, Pilot-Matias T 22 9 2017 Resistance characterization of hepatitis C virus genotype 2 from Japanese patients treated with ombitasvir and paritaprevir/ritonavir. J Med Virol doi:10.1002/jmv.24923. PMC6680211 28842997

[29] Zeuzem S, Dusheiko GM, Salupere R, Mangia A, Flisiak R, Hyland RH, Illeperuma A, Svarovskaia E, Brainard DM, Symonds WT, Subramanian GM, McHutchison JG, Weiland O, Reesink HW, Ferenci P, Hezode C, Esteban R 2014 Sofosbuvir and ribavirin in HCV genotypes 2 and 3. N Engl J Med 370:1993–2001. doi:10.1056/NEJMoa1316145. 24795201

[30] Kumada H, Suzuki Y, Ikeda K, Toyota J, Karino Y, Chayama K, Kawakami Y, Ido A, Yamamoto K, Takaguchi K, Izumi N, Koike K, Takehara T, Kawada N, Sata M, Miyagoshi H, Eley T, McPhee F, Damokosh A, Ishikawa H, Hughes E 2014 Daclatasvir plus asunaprevir for chronic HCV genotype 1b infection. Hepatology 59:2083–2091. doi:10.1002/hep.27113. 24604476 PMC4315868

[31] Akuta N, Sezaki H, Suzuki F, Fujiyama S, Kawamura Y, Hosaka T, Kobayashi M, Saitoh S, Suzuki Y, Arase Y, Ikeda K, Kumada H 2017 Ledipasvir plus sofosbuvir as salvage therapy for HCV genotype 1 failures to prior NS5A inhibitors regimens. J Med Virol 89:1248–1254. doi:10.1002/jmv.24767. 28079269

[32] Akuta N, Sezaki H, Suzuki F, Fujiyama S, Kawamura Y, Hosaka T, Kobayashi M, Saitoh S, Suzuki Y, Arase Y, Ikeda K, Kumada H 2017 Retreatment efficacy and predictors of ledipasvir plus sofosbuvir to HCV genotype 1 in Japan. J Med Virol 89:284–290. doi:10.1002/jmv.24617. 27357737

[33] Iio E, Shimada N, Takaguchi K, Senoh T, Eguchi Y, Atsukawa M, Tsubota A, Abe H, Kato K, Kusakabe A, Miyaki T, Matsuura K, Matsunami K, Shinkai N, Fujiwara K, Nojiri S, Tanaka Y 6 5 2017 Clinical evaluation of sofosbuvir/ledipasvir in patients with chronic hepatitis C genotype 1 with and without prior daclatasvir/asunaprevir therapy. Hepatol Res doi:10.1111/hepr.12898. 28332272

[34] Mizokami M, Dvory-Sobol H, Izumi N, Nishiguchi S, Doehle B, Svarovskaia ES, De-Oertel S, Knox S, Brainard DM, Miller MD, Mo H, Sakamoto N, Takehara T, Omata M 2016 Resistance analyses of Japanese hepatitis C-infected patients receiving sofosbuvir or ledipasvir/sofosbuvir containing regimens in phase 3 studies. J Viral Hepat 23:780–788. doi:10.1111/jvh.12549. 27196675

[35] Krishnan P, Schnell G, Tripathi R, Beyer J, Reisch T, Zhang X, Setze C, Rodrigues L Jr, Burroughs M, Redman R, Chayama K, Kumada H, Collins C, Pilot-Matias T 2016 Analysis of hepatitis C virus genotype 1b resistance variants in Japanese patients treated with paritaprevir-ritonavir and ombitasvir. Antimicrob Agents Chemother 60:1106–1113. doi:10.1128/AAC.02606-15. 26643326 PMC4750684

[36] McPhee F, Hernandez D, Zhou N, Ueland J, Yu F, Vellucci V, Huang X, Wang X, Ishikawa H, Karino Y, Kumada H 8 6 2017 Pooled analysis of HCV genotype 1 resistance-associated substitutions in NS5A, NS3 and NS5B pre- and post-treatment with 12 weeks of daclatasvir, asunaprevir and beclabuvir. Antivir Ther doi:10.3851/IMP3177. 28594332

[37] Lawitz EJ, Dvory-Sobol H, Doehle BP, Worth AS, McNally J, Brainard DM, Link JO, Miller MD, Mo H 2016 Clinical resistance to velpatasvir (GS-5816), a novel pan-genotypic inhibitor of the hepatitis C virus NS5A protein. Antimicrob Agents Chemother 60:5368–5378. doi:10.1128/AAC.00763-16. 27353271 PMC4997818

[38] Kai Y, Hikita H, Morishita N, Murai K, Nakabori T, Iio S, Hagiwara H, Imai Y, Tamura S, Tsutsui S, Naito M, Nishiuchi M, Kondo Y, Kato T, Suemizu H, Yamada R, Oze T, Yakushijin T, Hiramatsu N, Sakamori R, Tatsumi T, Takehara T 2017 Baseline quasispecies selection and novel mutations contribute to emerging resistance-associated substitutions in hepatitis C virus after direct-acting antiviral treatment. Sci Rep 7:41660. doi:10.1038/srep41660. 28134353 PMC5278351

[39] Itakura J, Kurosaki M, Hasebe C, Osaki Y, Joko K, Yagisawa H, Sakita S, Okushin H, Satou T, Hisai H, Abe T, Tsuji K, Tamada T, Kobashi H, Mitsuda A, Ide Y, Ogawa C, Tsuruta S, Takaguchi K, Murakawa M, Asahina Y, Enomoto N, Izumi N 2016 Complex pattern of resistance-associated substitutions of hepatitis C virus after daclatasvir/asunaprevir treatment failure. PLoS One 11:e0165339. doi:10.1371/journal.pone.0165339. 27776192 PMC5077083

[40] McPhee F, Hernandez D, Yu F, Ueland J, Monikowski A, Carifa A, Falk P, Wang C, Fridell R, Eley T, Zhou N, Gardiner D 2013 Resistance analysis of hepatitis C virus genotype 1 prior treatment null responders receiving daclatasvir and asunaprevir. Hepatology 58:902–911. doi:10.1002/hep.26388. 23504694

[41] Forns X, Lee SS, Valdes J, Lens S, Ghalib R, Aguilar H, Felizarta F, Hassanein T, Hinrichsen H, Rincon D, Morillas R, Zeuzem S, Horsmans Y, Nelson DR, Yu Y, Krishnan P, Lin CW, Kort JJ, Mensa FJ 2017 Glecaprevir plus pibrentasvir for chronic hepatitis C virus genotype 1, 2, 4, 5, or 6 infection in adults with compensated cirrhosis (EXPEDITION-1): a single-arm, open-label, multicentre phase 3 trial. Lancet Infect Dis 17:1062–1068. doi:10.1016/S1473-3099(17)30496-6. 28818546

[42] Kwo PY, Poordad F, Asatryan A, Wang S, Wyles DL, Hassanein T, Felizarta F, Sulkowski MS, Gane E, Maliakkal B, Overcash JS, Gordon SC, Muir AJ, Aguilar H, Agarwal K, Dore GJ, Lin CW, Liu R, Lovell SS, Ng TI, Kort J, Mensa FJ 2017 Glecaprevir and pibrentasvir yield high response rates in patients with HCV genotype 1-6 without cirrhosis. J Hepatol 67:263–271. doi:10.1016/j.jhep.2017.03.039. 28412293

[43] Poordad F, Felizarta F, Asatryan A, Sulkowski MS, Reindollar RW, Landis CS, Gordon SC, Flamm SL, Fried MW, Bernstein DE, Lin CW, Liu R, Lovell SS, Ng TI, Kort J, Mensa FJ 2017 Glecaprevir and pibrentasvir for 12 weeks for hepatitis C virus genotype 1 infection and prior direct-acting antiviral treatment. Hepatology 66:389–397. doi:10.1002/hep.29081. 28128852 PMC5573922

[44] Koletzki D, Dumont S, Vermeiren H, Fevery B, De Smet P, Stuyver LJ 2010 Development and evaluation of an automated hepatitis C virus NS5B sequence-based subtyping assay. Clin Chem Lab Med 48:1095–1102. doi:10.1515/CCLM.2010.236. 20578969

[45] Murphy DG, Willems B, Deschenes M, Hilzenrat N, Mousseau R, Sabbah S 2007 Use of sequence analysis of the NS5B region for routine genotyping of hepatitis C virus with reference to C/E1 and 5′ untranslated region sequences. J Clin Microbiol 45:1102–1112. doi:10.1128/JCM.02366-06. 17287328 PMC1865836

[46] Katoh K, Asimenos G, Toh H 2009 Multiple alignment of DNA sequences with MAFFT. Methods Mol Biol 537:39–64. doi:10.1007/978-1-59745-251-9_3. 19378139

[47] Saitou N, Nei M 1987 The neighbor-joining method: a new method for reconstructing phylogenetic trees. Mol Biol Evol 4:406–425. 3447015 10.1093/oxfordjournals.molbev.a040454

[48] Studier JA, Keppler KJ 1988 A note on the neighbor-joining algorithm of Saitou and Nei. Mol Biol Evol 5:729–731. 3221794 10.1093/oxfordjournals.molbev.a040527

