# Supplementary material for: Integrated Resistance Analysis of CERTAIN-1 and CERTAIN-2 Studies in Hepatitis C Virus-Infected Patients Receiving Glecaprevir and Pibrentasvir in Japan
Source: Antimicrob Agents Chemother. 2018 Jan 25;62(2):e02217-17. doi: 10.1128/AAC.02217-17 (PMC5786793; doi:10.1128/AAC.02217-17)
Supplement: Supplemental material [file supp_62_2_e02217-17__index.html]

Supplemental material 

# Integrated Resistance Analysis of CERTAIN-1 and CERTAIN-2 Studies in Hepatitis C Virus-Infected Patients Receiving Glecaprevir and Pibrentasvir in Japan

## Supplemental material

- Supplemental file 1 -

  Supplemental Tables S1 and S2 and Figures S1 and S2

  PDF, 115K
