# Supplementary material for: Integrated Resistance Analysis of CERTAIN-1 and CERTAIN-2 Studies in Hepatitis C Virus-Infected Patients Receiving Glecaprevir and Pibrentasvir in Japan
Source: Antimicrob Agents Chemother. 2018 Jan 25;62(2):e02217-17. doi: 10.1128/AAC.02217-17 (PMC5786793; doi:10.1128/AAC.02217-17)
Supplement: Supplemental material [file AAC.02217-17_zac002186878s1.pdf]

Supplemental Table 1. Baseline Substitutions in NS3 and NS5A In DAA-Experienced GT1b-Infected Patients

Prior DAA treatments[a]	Months since failure with prior DAA regimen	HCV GT[b]	NS3 Substitutions at Baseline[b,c]	NS5A Substitutions at Baseline[b,c]
Asunaprevir + Daclatasvir	12.7	1b*	D168V	P32L (<15%), P32deletion
Asunaprevir + Daclatasvir	12.3	1b	None	L31F + P32deletion
Asunaprevir + Daclatasvir	8.5	1b	D168E	L31M + Y93H
Ombitasvir + Paritaprevir; Asunaprevir + Daclatasvir	13.8	1b*	None	L31M + Y93H
Asunaprevir + Daclatasvir	13.1	1b	D168E	L31I (<15%), L31V, Y93H
Asunaprevir + Daclatasvir	10.5	1b*	D168E	L31V + Y93H
Asunaprevir + Daclatasvir	10.6	1b	None	R30H + L31I + Y93H, R30Q (<15%), P58S (<15%)
Asunaprevir + Daclatasvir	12.5	1b	None	Q24K + L28I + R30L + L31I, P58L
Telaprevir; Simeprevir; Asunaprevir + Daclatasvir	14.5	1b	D168E	L31M, L31V (<15%)
Telaprevir; Asunaprevir + Daclatasvir	12.5	1b	None	Q24K + L28M + R30H + L31M
Telaprevir; Asunaprevir + Daclatasvir	11.9	1b	None	Q24K + L28M + R30M + L31V + Y93H
Asunaprevir + Daclatasvir	6.7	1b*	None	L31V + Y93H
Asunaprevir + Daclatasvir	11.2	1b	D168E	L31M/V + Y93H
Asunaprevir + Daclatasvir	6.0	1b	NA	L31M + Y93H
Asunaprevir + Daclatasvir	16.1	1b	None	Q24K + L28V + L31M, R30H, R30Q (<15%)
Telaprevir; Asunaprevir + Daclatasvir	6.4	1b	D168E	L31V + Y93H
Asunaprevir + Daclatasvir	9.9	1b	None	R30L + L31M + A92K
Asunaprevir + Daclatasvir	11.5	1b	None	None
Asunaprevir + Daclatasvir	13.6	1b	D168E	L31V (<15%), L31M + Y93H
Asunaprevir + Daclatasvir	12.9	1b	D168E	L31M + Y93H
Asunaprevir + Daclatasvir	10.8	1b	None	L31M, L31V, Y93H
Asunaprevir + Daclatasvir	11.3	1b	None	L31I + A92T + Y93H
Asunaprevir + Daclatasvir	8.5	1b	D168E	L31F
Simeprevir; Asunaprevir + Daclatasvir	6.4	1b	D168E	L31V + Y93H
Asunaprevir + Daclatasvir	11.1	1b	D168A (<15%)	Q24K + L28M + R30H + Y93F
Simeprevir; Asunaprevir + Daclatasvir	7.8	1b	D168V	R30Q + L31M + Y93H
Asunaprevir + Daclatasvir	6.4	1b	D168T	L31V + Y93H
Asunaprevir + Daclatasvir	9.6	1b	D168E	Q24K + L28M + R30Q + A92T + Y93S
Asunaprevir + Daclatasvir	11.8	1b	None	R30Q + L31F + Y93H
Simeprevir; Asunaprevir + Daclatasvir	4.7	1b	D168V	Q24K + L31I, L28M/T, A92T, Y93H
Simeprevir	19.3	1b	None	A92T
Simeprevir	11.7	1b	None	Q24K + L28M + R30L + P58L

a. Prior treatment with IFN or RBV are not listed.

b. Patients who experienced virologic failure are indicated by shading. * indicates patients with compensated cirrhosis.

c. Baseline substitutions detected at the 2% but not at the 15% threshold are indicated as "(<15%)". For samples with multiple substitutions within a target, if individual substitutions were detected at ≥ 90% prevalence, they are considered to be linked and denoted by "+", whereas if one or more of the substitutions was detected at <90% prevalence, the variants are separated by a comma.

d. Amino acid positions for NS3/4A protease inhibitor class included in the analysis: 155, 156, and 168. Amino acid positions for NS5A inhibitor class included in the analysis: 24, 28, 29, 30, 31, 32, 58, 92, and 93.

Supplemental Table 2. Comparison of SVR_{12} rates in DAA-experienced Patients with or without Baseline Substitutions at 15% Detection Threshold

Target	HCV GT	Baseline Polymorphism	G/P 12 Weeks % SVR_{12} (n/N)		
			With BP	Without BP	P Value[a]
NS3	1b	Y56F	83.3 (5/6)	96.0 (24/25)	0.36
		Q80K/L/R	90.9 (10/11)	95.0 (19/20)	1.0
		V107I	100 (1/1)	93.3 (28/30)	1.0
		S122G/T	100 (13/13)	88.9 (16/18)	0.50
		D168E/T/V	93.3 (14/15)	93.8 (15/16)	1.0
		V170I	93.3 (14/15)	93.8 (15/16)	1.0
		Any	92.6 (25/27)	100 (4/4)	1.0
NS5A		Q24K	100 (8/8)	91.7 (22/24)	1.0
		L28I/M/T/V	100 (8/8)	91.7 (22/24)	1.0
		R30H/L/M/Q	100 (11/11)	90.5 (19/21)	0.53
		L31F/I/M/V	96.2 (25/26)	83.3 (5/6)	0.35
		P32deletion	(0/2)	100 (30/30)	0.002*
		Q54H/L	100 (9/9)	91.3 (21/23)	1.0
		P58L	100 (2/2)	93.3 (28/30)	1.0
		Q62E/K/P	100 (3/3)	93.1 (27/29)	1.0
		A92K/T	100 (5/5)	92.6 (25/27)	1.0
		Y93F/H/S	100 (21/21)	81.8 (9/11)	0.11
		Any	93.5 (29/31)	100 (1/1)	1.0
NS3	2a	Any	--	100 (1/1)	--
NS5A		T24A	100 (1/1)	--	--
		L31M	100 (1/1)	--	--
		Any	100 (1/1)	--	--

a. P value by Fisher's exact test. * indicates value is statistically significant at 0.01 level.

Supplemental Figure 1. Prevalence of Baseline Polymorphisms in NS3 in DAA treatment-naïve GT1b-infected Patients in Japan, North America, Europe, and ROW

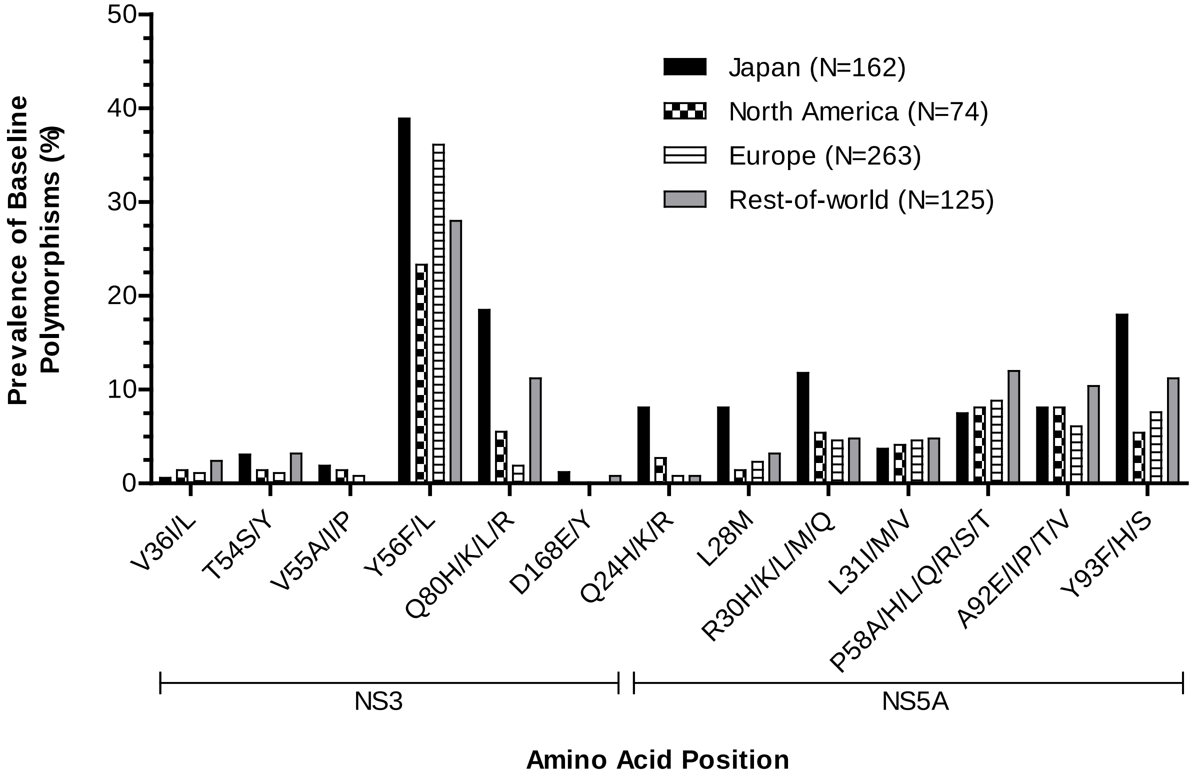

Prevalence of baseline polymorphisms at 15% detection threshold relative to GT1b-Con1 reference sequence at amino acid positions of interest for the NS3/4A protease inhibitor or NS5A inhibitor class is shown. When defining geographic regions, sites in Canada, Mexico, Puerto Rico and the United States were grouped under North America; sites in Austria, Belgium, France, Germany, Greece, Hungary, Italy, Lithuania, Poland, Portugal, Romania, Spain, Sweden, Switzerland, and United Kingdom were grouped under Europe; sites in Australia, Chile, Israel, Korea, New Zealand, South Africa, and Taiwan were grouped together as rest of world (ROW).

Supplemental Figure 2. Prevalence of baseline polymorphisms in NS3 and NS5A in DAA treatment-naïve GT2-infected patients in Japan, North America, Europe, and ROW.

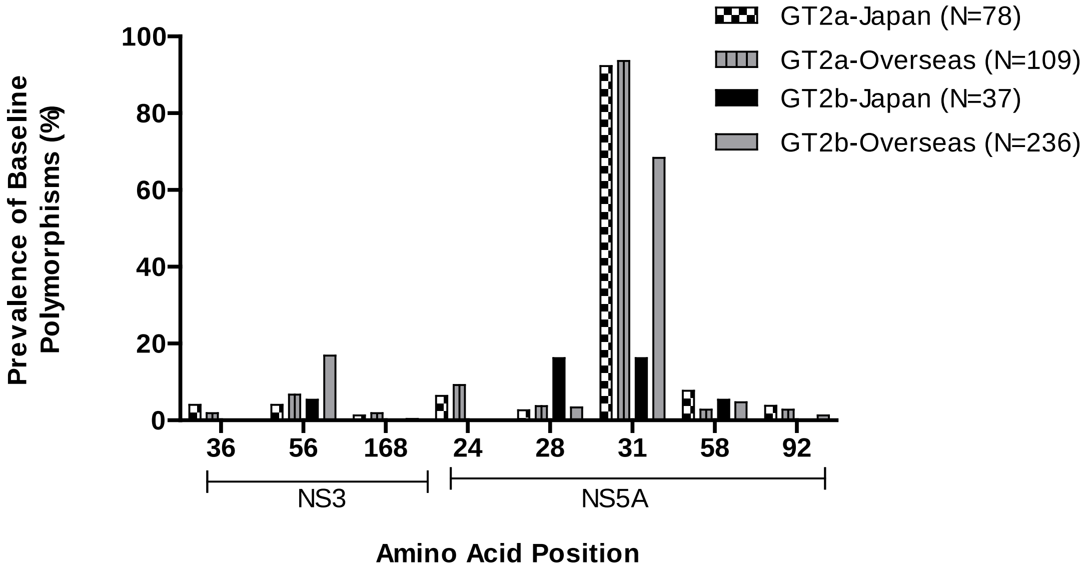

GT	Strain	Amino Acids in Reference Strain at Positions of Interest in NS3								
		36	43	54	55	56	80	155	156	168
2a	JFH-1	L	F	T	V	Y	G	R	A	D
2b	HC-J8	L	F	T	V	Y	G	R	A	D

GT	Strain	Amino Acids in Reference Strain at Positions of Interest in NS5A							
		24	28	30	31	32	58	92	93
2a	JFH-1	T	F	K	L	P	P	C	Y
2b	HC-J8	S	L	K	M	P	P	C	Y

Prevalence of baseline polymorphisms at 15% detection threshold relative to GT2a-JFH-1 or GT2b-HC-J8 reference sequence at amino acid positions of interest for the NS3/4A protease or NS5A inhibitor class is shown. Overseas regions include North America, Europe and ROW. Amino acids at positions important for PI and NS5A inhibitor class are shown in the inset table. Baseline polymorphisms were not detected at positions 43, 54, 55, 155, 156 in NS3, or 30, 32, 93 in NS5A.
